# Molecular Population Genetics of Human *CYP3A* Locus: Signatures of Positive Selection and Implications for Evolutionary Environmental Medicine

**DOI:** 10.1289/ehp.0800528

**Published:** 2009-06-18

**Authors:** Xiaoping Chen, Haijian Wang, Gangqiao Zhou, Xiumei Zhang, Xiaojia Dong, Lianteng Zhi, Li Jin, Fuchu He

**Affiliations:** 1 State Key Laboratory of Proteomics, Beijing Proteome Research Center, Beijing Institute of Radiation Medicine, Chinese National Human Genome Center at Beijing, Beijing, China; 2 Department of Pharmacology, School of Pharmaceutical Science, Central South University, Changsha, Hunan, China; 3 Laboratory of Systems Biology, State Key Laboratory of Genetic Engineering and MOE Key Laboratory of Contemporary Anthropology, School of Life Sciences and Institutes of Biomedical Sciences, Fudan University, Shanghai, China; 4 The Simons Center for Systems Biology, School of Natural Sciences, Institute for Advanced Study, Princeton, New Jersey, USA

**Keywords:** CYP3A, environmental genomics, genetic polymorphism, positive selection

## Abstract

**Background:**

The human *CYP3A* gene cluster codes for cytochrome P450 (CYP) subfamily enzymes that catalyze the metabolism of various exogenous and endogenous chemicals and is an obvious candidate for evolutionary and environmental genomic study. Functional variants in the *CYP3A* locus may have undergone a selective sweep in response to various environmental conditions.

**Objective:**

The goal of this study was to profile the allelic structure across the human *CYP3A* locus and investigate natural selection on that locus.

**Methods:**

From the *CYP3A* locus spanning 231 kb, we resequenced 54 genomic DNA fragments (a total of 43,675 bases) spanning four genes (*CYP3A4*, *CYP3A5*, *CYP3A7*, and *CYP3A43*) and two pseudogenes (*CYP3AP1* and *CYP3AP2*), and randomly selected intergenic regions at the *CYP3A* locus in Africans (24 individuals), Caucasians (24 individuals), and Chinese (29 individuals). We comprehensively investigated the nucleotide diversity and haplotype structure and examined the possible role of natural selection in shaping the sequence variation throughout the gene cluster.

**Results:**

Neutrality tests with Tajima’s *D*, Fu and Li’s D* and F*, and Fay and Wu’s *H* indicated possible roles of positive selection on the entire *CYP3A* locus in non-Africans. Sliding-window analyses of nucleotide diversity and frequency spectrum, as well as haplotype diversity and phylogenetically inferred haplotype structure, revealed that *CYP3A4* and *CYP3A7* had recently undergone or were undergoing a selective sweep in all three populations, whereas *CYP3A43* and *CYP3A5* were undergoing a selective sweep in non-Africans and Caucasians, respectively.

**Conclusion:**

The refined allelic architecture and selection spectrum for the human *CYP3A* locus highlight that evolutionary dynamics of molecular adaptation may underlie the phenotypic variation of the xenobiotic disposition system and varied predisposition to complex disorders in which xenobiotics play a role.

A key event in human population history is the dispersal of early humans from Africa to other parts of the world with different climates, pathogens, sources of food and xenobiotic exposure, and the dynamic adaptation to the evolving environments (Jin and Su 2000). Current phenotypic differences between individuals/groups could be due partly to functional polymorphisms that facilitated survival in the ancestral populations (Di Rienzo and Hudson 2005). Resolving the underlying allelic architectures of environmental response and searching for their molecular adaptation to selective forces have been attractive indirect strategies for implementation and interpretation of genetic analysis of environmental response and complex disorders (Bamshad and Wooding 2003; Sabeti et al. 2007).

Members of the cytochrome P450 (CYP) 3A family are among the most important CYP enzymes in humans. They metabolize various endogenous and exogenous chemicals, such as clinically important drugs, environmental carcinogens, cholesterol, steroids, and other lipids (Wojnowski 2004). Members of the CYP3A family are expressed in organs primarily associated with xenobiotic and hormone disposition, such as the liver and gastrointestinal tract (Wojnowski 2004). Four functional CYP3A enzymes—CYP3A4, CYP3A5, CYP3A7, and CYP3A43—have been identified in humans. CYP3A4 is most abundant in adult liver and intestine and is the major enzyme involved in xenobiotic and drug metabolism (Fujita 2004). CYP3A5 is the predominant form in the kidney (Givens et al. 2003). CYP3A7, a fetoplacental enzyme found only in humans, is expressed mainly in fetal liver (Leeder et al. 2005) and extrahepatic tissues such as endometrium and placenta (Burk et al. 2002; Schuetz et al. 1993). The more recently cloned CYP3A43 is expressed predominantly in prostate and testis (Gellner et al. 2001). Interindividual variation in CYP3A activity up to 10-fold has been observed (Dorne et al. 2003). Because CYP3A activity is a major determinant of drug response and may be associated with risk for cancers such as breast and prostate cancer (Keshava et al. 2004), the identification of sequence variants at the *CYP3A* locus and functional characterization of their clinical relevance have been of long-standing interest in pharmacogenetics and toxicogenetics.

The human *CYP3A* gene cluster resides in a 231-kb region on chromosome 7q22 and consists of four genes and two pseudogenes, arranged in the order of *CYP3A5*, *CYP3A5P1*, *CYP3A7*, *CYP3A5P2*, *CYP3A4*, and *CYP3A43*, from centromere to telo mere (Finta and Zaphiropoulos 2000) (Figure 1). *CYP3A43* is in the opposite orientation from all other *CYP3A* genes. Each intact gene encodes a protein consisting of 503 amino acids. Finta and Zaphiropoulos (2000) suggested that the locus arose through duplication of an ancestral *CYP3A* cassette of 40–50 kb. Many genetic variants have been identified in this locus and are available online (http://www.imm.ki.se/CYPalleles/); some functional variants for *CYP3A* have been reported, such as the CYP3A5*3, which results in an incorrectly spliced mRNA and a truncated nonfunctional protein (Kuehl et al. 2001).

Parallel to exploring phenotypic effects and clinical relevance of *CYP3A* genetic polymorphisms, investigating molecular adaptations to the environment is an intriguing complementary strategy for pharmacogenetic and toxicogenetic studies. The *CYP3A4* and *CYP3A5* genes have a strong haplotype structure at varying frequencies across ethnic groups (Thompson et al. 2004). Using a comparative genomics approach and sequence-based neutrality test, Thompson et al. (2004) reported evidence of positive selection on the derived allele of the functional CYP3A5*3 in non-African populations and significant correlation of its allelic frequency with distance from the equator. In a genotype-based investigation on the *CYP3A* locus, Schirmer et al. (2006) proposed that negative natural selection acted primarily toward the elimination in non-African populations of the ancestral CYP3A4*1B allele rather than toward the reduction of CYP3A5 expression described by Thompson et al. (2004). It should be noted that these population genetics studies were based on a small fraction of sequence information or on only limited data of genotypes of single-nucleotide polymorphisms (SNPs), and both had limited coverage of the 230-kb genomic sequence of *CYP3A* locus. Therefore, the refined molecular targets of natural selection on the complete *CYP3A* locus and its evolutionary dynamics with respect to geographically and temporally fluctuating environments in our demographic history have not yet been explicitly pinpointed.

To further identify genetic variations in the human *CYP3A* locus and assess the effect of natural selection on the pattern of nucleotide diversity at this locus, we resequenced the human *CYP3A* locus in three populations and addressed evolutionary population genetics on the gene cluster.

## Materials and Methods

### Subjects and samples

We obtained human genomic DNA samples from two sources: *a* ) we extracted genomic DNA samples from venous blood for 29 unrelated healthy Chinese men (average age, 20 ± 2 years) who were chosen from the sample collection constructed for the Chinese Human Genome Diversity Project through a coordinated effort of several institutes (Chu et al. 1998); and *b*) we used DNA samples of 24 Caucasians (11 females, 13 males), 24 Africans (9 females, 15 males), and three apes [i.e., one common chimpanzee (*Pan troglodytes*), one lowland gorilla (*Gorilla gorilla*), and one orangutan (*Pongo pygmaeus*)] provided by Coriel Cell Repositories (Camden, NJ, USA). Written informed consent was obtained from all the Chinese subjects. This study was performed with the approval of the Ethical Committee of Chinese National Human Genome Center at Beijing.

### Sequencing strategy

**Figure 1:**
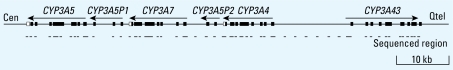
Genomic structure of genes and pseudogenes at the *CYP3A* locus. Abbreviations: Cen, centromere; Qtel, telomere. Exons for genes and pseudogenes are shown as boxes; arrows indicate transcriptional orientation; and broken lines indicate resequenced regions.

A schematic representation of genes and pseudogenes at the *CYP3A* locus and the resequenced regions is shown in Figure 1. We screened 54 genomic DNA fragments totaling 43,675 bases. The resequenced regions included nearly all exons (except exon 11 of *CYP3A7* because of difficulty in sequencing the highly homologous sequences) and relevant exon–intron boundaries of four genes (*CYP3A43*, *CYP3A4*, *CYP3A7*, and *CYP3A5*) and two pseudogenes (*CYP3AP1* and *CYP3AP2*) and approximately 2.5-kb promoter regions of the four intact genes. We also resequenced four randomly selected segments equally distributed across the *CYP3A43*–*CYP3A4* gene interval and a fragment in *CYP3AP2*–*CYP3A7* interval, with an average length of 1.1 kb.

### Identification of polymorphisms and divergences

We identified polymorphisms by sequencing polymerase chain reaction (PCR) products from both ends. Long-range PCR (L-PCR) amplifications were performed first with primers specific for the target DNA regions to increase specificity [GenBank accession no. NG_000004.2 (National Center for Biotechnology Information 2009)]. DNA sequencing was performed using overlapping primers that covered the overall L-PCR fragments. We confirmed positions and individual genotypes of the variations by resequencing from the opposite strand or from overlapped sequencing. Fixed differences or divergences between humans and apes were inferred by aligning the resequenced sequences of humans with those of the three apes; we considered the alleles that occurred in the apes to be the ancestral alleles for polymorphic sites. Details regarding PCR and sequencing conditions, as well as PCR primers, are available on request. As a measure of quality control, sequence segments of individuals presenting singletons or ambiguous polymorphisms were reamplified and resequenced. We assessed SNP data validity by repeating 10% of the assays. The error rate was relatively low (1.2%).

### Data analysis

We performed tests of fitness to the Hardy-Weinberg equilibrium for each polymorphic site using LDA software (Ding et al. 2003). We used the false discovery rate method to correct for multiple testing using Q-VALUE in R (Dabney and Storey 2002) as described by Storey and Tibshirani (2003). Three measures of nucleotide diversity were assessed: average nucleotide diversity (π), a statistic based on the average number of pairwise sequence differences and influenced mostly by intermediate-frequency variants (Tajima 1989); Watterson’s θ_W_, which is based on the number of segregating sites and influenced mostly by low-frequency variations, theoretically equal to the neutral mutation parameter *4N_e_*m (Watterson 1975); and θ_H_ (Fay and Wu 2000), a summary that gives more weight to high frequency–derived alleles. To test whether the frequency spectrum of polymorphisms conformed to the standard neutral model, we calculated the values of four test statistics: Tajima’s *D*, which considers the difference between π and θ_W_ (Tajima 1989); Fu and Li’s F* and D*, which compare the number of singletons with the number of nonsingletons (Fu and Li 1993); and Fay and Wu’s *H*, which compared the high-frequency with intermediate frequency–derived variants (Fay and Wu 2000). Significant values for these test statistics were estimated from 10^4^ coalescent simulations of a Wright-Fisher equilibrium model that conditioned on a constant sample size and the level of polymorphism as the observed data (Kaplan et al. 1991). We also performed the Hudson–Kreitman–Aguade (HKA) test to compare the diversity patterns in the sequences observed in our study and those observed in *DMD* intron 44 (Nachman and Crowell 2000). All neutrality tests and coalescent simulations were performed using DnaSP software, version 4.0 (Rozas et al. 2003).

We calculated an unbiased small-sample estimator of pairwise populations *F*_ST_ (F-statistic/fixation index), a measure of allele frequency difference among populations and an estimate of interpopulation genetic differentiation, as described previously (Weir and Cockerham 1984). A bootstrapping method (1,000 bootstrap samples) was used to test the statistical significance of *F*_ST_ in each pairwise comparison between populations. Lewontin’s D′ and *r*^2^ were applied to evaluate the pairwise linkage disequilibrium (LD) between biallelic polymorphisms. We used Fisher’s exact test to determine the statistical significance of pairwise LD, followed by false discovery rate correction for multiple testing. We used GOLD software (Center for Statistical Genetics 2009) as described by Abecasis and Cookson 2000) to draw the LD maps.

Haplotypes were constructed using the Phase 2.0 program (Stephens and Donnelly 2003). We then partitioned haplotype blocks with HaploBlockFinder (Zhang 2004) as described by Zhang and Jin (2003). The mutational relationships among haplotypes in *CYP3A43* and *CYP3A5* were shown by constructing minimum-spanning trees (MSTs), with the common chimpanzee as an out-group species. We calculated the networks by reduced median algorithm for *CYP3A5* and median jointing algorithm for *CYP3A43* implicated in the Network 4.1 package (Fluxus Technology 2008) as described by Bandelt et al. (1999).

## Results

### Data summary and sequence variations

We sampled 77 unrelated human individuals, including 24 Africans, 24 Europeans, and 29 Asians. The surveyed sequences spanned 43,675 kb and contained almost all of the coding sequences of *CYP3A4*, *CYP3A5*, *CYP3A7*, and *CYP3A43*. We observed a total of 167 segregating sites (including 165 biallelic SNPs and two biallelic indel polymorphisms) in the human DNA samples [see Supplemental Material, Table 1, available online (doi:10.1289/ehp.0800528.S1 via http://dx.doi.org/)], yielding an average density of one SNP per 262 bp. Of the variants identified, 11 were nonsynonymous, 2 resulted in a splicing defect, 1 was a frame shift mutation, and 8were synonymous.

Sequence divergence between human and chimpanzee was 0.95–0.97% for the total locus in subpopulations, which was comparable with the genome-wide average of 1.24% (Ebersberger et al. 2002). Nucleotide diversity was low in both Chinese and Caucasians: 1.6 × 10^−4^ and 1.4 × 10^−4^, respectively, for π; 2.8 × 10^−4^ and 3.1 × 10^−4^, respectively, for θ_W_ (Table 1). When the *F*_ST_ for three-way population comparisons were estimated with polymorphisms site-by-site, 11 *F*_ST_ values (ranging from 0.30 to 0.50) fell into the upper 0.05 tail of the empirical genomewide distributions estimated previously (Bowcock et al. 1991, Storz et al. 2004) [see Supplemental Material, Table 1 (doi:10.1289/ehp.0800528. S1)]. Comparison of *F*_ST_ for Africans and non-Africans using the empirical genome-wide *F*_ST_ distribution constructed with similar sets of pooled non-African and African samples (Fullerton et al. 2002) showed that estimated *F*_ST_ values for five variants (T147767C, G164751T, T165611C, A169228G, and A260167G) were extremely high (≥ 0.40; data not shown) and fell above the 95th percentile of the empirical distribution.

### Selective neutrality tests

**Figure 2:**
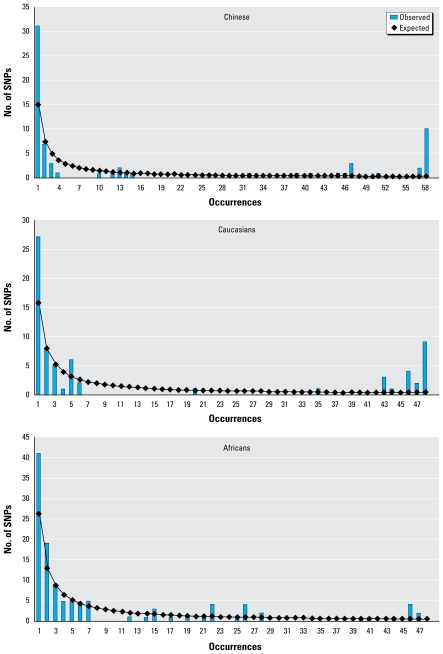
Frequency spectrum of derived alleles at the *CYP3A* locus in Chinese, Caucasians, and Africans. The expected frequency spectrum has been reported by Watterson (1975); at the neutral equilibrium, the expected number of SNP sites at which the derived allele is present *i* times in the sample is given by 4*Nv/i*, where *N* and *v* are the effective population size and mutation rate, respectively. The formula to estimate 4*Nv* is the number of observed segregating sites divided by (1 + 1/2 + 1/3 + . . . + 1/*n*−1), where *n* is the number of chromosomes in each population.

Frequency spectra of the derived alleles for the three populations are shown in Figure 2. Notably, we observed an excess of singletons and nearly fixed SNPs (derived allelic frequencies > 80%). Consistently, Tajima’s *D*, and Fu and Li’s F* and D* deviated significantly from expectations under neutrality in all three populations examined. When individual populations were considered, *D* and F* values in both Chinese and Caucasians were significant (*p* < 0.05), and D* was significant in Chinese (*p* < 0.05). We also observed a statistically significant *H* value in Caucasians (*p* = 0.046). Results of the HKA test indicated that the nucleotide diversity patterns in the entire surveyed sequences were significantly different from those in the neutral *DMD* intron 44 in all three subpopulations (*p* < 0.05).

**Figure 3:**
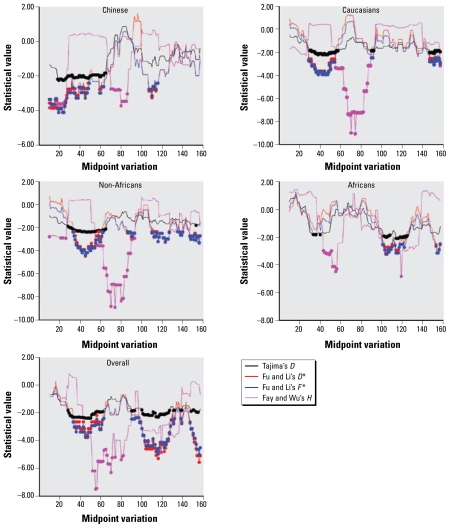
Sliding-window neutrality tests of *CYP3A* locus. Each window contains 20 variations, with a step size of one variation. The midpoint variations at the 20th, 40th, 60th, 80th, 100th, 120th, 140th, and 160th sites are T88726C, T116513G, C147742T, A169228G, G186731A, A211137T, T251839A, and T270403C, respectively (as shown in Table 1). Circles indicate significant values for corresponding neutrality tests.

We performed sliding-window analyses for the neutrality tests, with each window containing 20 variations and with a step size of one variation. The values of the neutrality statistics varied widely across the locus in all three populations and in subpopulations (Figure 3). We observed significantly negative *D*, D*, F*, and *H* values simultaneously in two regions in all the three populations. The first region covered the 40.5-kb sequence from the *CYP3A43*–*CYP3A4* interval to *CYP3A4* intron 6, or between SNPs C122872T and T163355G. The second region covered the 25.7-kb sequence from the *CYP3A5P2*–*CYP3A7* interval to *CYP3A7* exon 7, or between SNPs G191479A and A217213G. In addition, significantly negative values for all four test statistics were also observed in the region from *CYP3A43* intron 10 to *CYP3A43* 5′-flank region (between SNPs A73018G and G106490T) in Chinese, and in the region from *CYP3A5* exon 2 to *CYP3A5* 3′ untranslated region (between SNPs C256880T and C284792T) in Caucasians.

### Linkage disequilibrium

**Figure 4:**
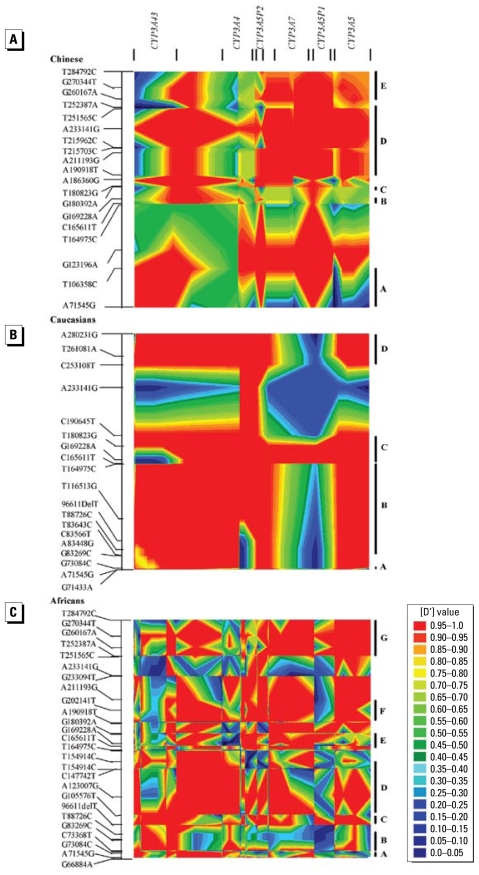
LD structures at the *CYP3A* locus in Chinese (*A*), Caucasians (*B*), and Africans (*C*) as indicated by pairwise LD between variations, with minor allele frequency > 5%, measured by |D’|. Haplotype blocks partitioned in each population are also shown to the right of corresponding LD structures.

When 24 SNPs shared by all three subpopulations were considered, mean pairwise |D′| values were significantly higher in Chinese (*p* = 0.001) and Caucasians (*p* < 0.001) compared with Africans: values were 0.93, 0.86, and 0.74, respectively. The Africans had far fewer SNP pairs with *r*^2^ ≥ 0.5 compared with either Chinese or Caucasians (6.5%, 25.0%, and 22.8%, respectively). Five SNPs in *CYP3A43* (G83269C, C83448T, A83566G, C83448T, and T88726C) were in complete LD with each other in all three populations. These five SNPs were also in significant LD with both the nonsynonymous substitution CYP3A43*3 and the frame shift mutation CYP3A43*2A in non-Africans. The patterns of LD structure in each population (Figure 4), delineated with polymorphisms with minor allelic frequency > 5% in individual populations, also showed remarkable differences in extent and strength of LD across the entire locus between Africans and non-Africans. When the threshold of |D′| value was set at 0.8, we observed five, four, and seven LD blocks in Chinese, Caucasians, and Africans, respectively. The longest blocks spanned 60.5 kb in Chinese (block D), 81.7 kb in Caucasians (block B), and 49.3 kb in Africans (block D) (Figure 4).

### Haplotype distribution

**Figure 5:**
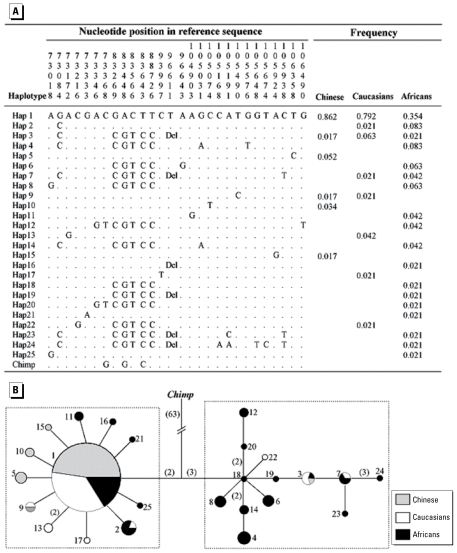
Haplotype in the region between SNPs A73018G and G106490T in *CYP3A43* and MST of the haplotypes. (*A*) Haplotypes and their estimated frequencies in each population, including the ancestral haplotype in chimpanzee (Chimp). (*B* ) MST of the haplotypes in this region: one main cluster with only one common haplotype (left) and one minor cluster containing haplotypes of low or intermediate frequency (right). The size of each node is proportional to haplotype frequency in all three populations, and the frequency of each haplotype within each subpopulation is indicated by various shades within each node. Branches represent one nucleotide substitution, unless noted in parentheses.

Because all four neutrality tests indicated that nucleo tide diversity patterns in the regions between SNPs A73018G and G106490T in *CYP3A43* and between SNPs C256880T and C284792T in *CYP3A5* deviated significantly from expected under neutrality in non-Africans, we constructed the haplotypes in these regions of *CYP3A43* and *CYP3A5* and depicted their mutational relationships by constructing MSTs rooted with the chimpanzee [Figure 5; see also Supplemental Material, Figure 1 (doi:10.1289/ehp.0800528.S1)]. The haplotype MSTs showed two clusters of haplotypes for both regions, one main cluster dominated with only one common haplotype (Figure 5B, left) and one minor cluster scattered with haplotypes with low or intermediate frequencies (Figure 5B, right). The haplotype clusters at the *CYP3A43* locus were separated by five nucleotides at positions 83269, 83448, 83566, 83643, and 88726, with high frequency–derived alleles at positions 83448 and 83643 (Figure 5B). The haplotype clusters at the *CYP3A5* locus were separated by two high frequency–derived alleles at positions 260167 and 284792 [see Supplemental Material, Figure 1 (doi:10.1289/ehp.0800528.S1)]. The haplotype diversity test showed that haplotype diversity in the *CYP3A43* region deviated obviously from that expected under neutrality in Chinese and Caucasians or non-Africans, and haplotype diversity in *CYP3A5* deviated significantly from that expected under neutrality in Caucasians (Table 2).

## Discussion and Conclusion

Several lines of evidence indicate signatures of natural selection on *CYP3A4* and *CYP3A7* in all three populations in our study. First of all, we observed extremely low nucleotide diversities at both gene loci in all three populations. The π values were obviously much lower than the genome-wide average for autosomes (7.7 × 10^−4^), across chromosome 7 (7.6 × 10^−4^) (Stephens et al. 2001), and in coding regions for autosomal genes (3.4 × 10^−4^) (Sachidanandam et al. 2001). Second, significantly negative values for all four statistics (Tajima’s *D*, Fu and Li’s D* and F*, and Fay and Wu’s *H*) were observed in *CYP3A4* and *CYP3A7*. Third, the HKA test also indicated a local reduction in variability relative to divergence at the *CYP3A* locus compared with the neutral *DMD* intron 44 (Nachman and Crowell 2000).

In agreement with our findings, Qiu et al. (2008) also detected particularly strong recent positive selections on protein-coding sequences of human *CYP3A4* and *CYP3A7* after the split of the chimpanzee and human lineages or early in hominoid evolution. In a recent phylogenetic investigation, Zawaira et al. (2008) also showed that the Gotoh’s substrate recognition sites of human CYP3A is associated with the sites under adaptive evolution. CYP3A is involved in the metabolism of foreign compounds, such as naturally occurring flavonoids, diterpenoids in germander, pyrrolizidine alkaloids (e.g., echimidine and jacobine), and herbal constituents (Zhou et al. 2007). CYP3A also normally metabolizes food-derived activators of pregnane X receptor and/or constitutive androstane receptor, thereby regulating the expression of a range of detoxifying systems in the liver (van Waterschoot et al. 2009). Therefore, molecular adaptation to the evolving environment is possible for the *CYP3A* locus or individual genes in this locus.

In the present study, we observed that the 40.5-kb region from the *CYP3A4* 5′-flanking region to *CYP3A4* intron 6 was under a recent positive selection in human samples overall. Thompson et al. (2004) also observed an excess of rare variants, as well as a low number of polymorphisms, in the *CYP3A4* gene in non-Africans but not Africans. Schirmer et al. (2006) observed that the region centromeric of the ancestral CYP3A4*1B allele (which is correlated with moderately increased expression of *CYP3A4* mRNA and protein and increased activity) exhibits “high haplotype homozygosity in European Caucasians as oppose d to African Americans.” The slight difference between our study and that of Thompson et al. (2004) may be due to differences in data analysis, as all the neutrality tests were performed on the assumption of absence of genetic recombination across the locus. However, recombination at the *CYP3A* locus is possible, as indicated by LD structure in the present study. Neutrality tests that take recombination into account, such as the sliding-window analysis, may be more appropriate. Of course, it is still difficult to pinpoint the evolutionary driving force shaping the nucleotide diversity pattern of *CYP3A4* in our study. In spite of its role in the metabolism of xenobiotics, CYP3A4 also metabolizes endogenous chemicals such as the eicosanoid metabolite endocannabinoid anandamide (Snider et al. 2007) and uroporphyrin (Franklin et al. 2000); factors that lead to fluctuation of these chemicals might be one of selective forces for human *CYP3A4.*

CYP3A7 shows distinctly high catalytic activities for the 16α-hydroxylation of steroids with a C17-keto group, such as estrone (Lee et al. 2003) and dehydroepiandrosterone (Ohmori et al. 1998), and plays a role in estriol synthesis (Siiteri and MacDonald 1963) and retinoic acid metabolism (Marill et al. 2002). Leeder et al. (2005) observed extremely low expression of CYP3A7 in livers of anencephalic human fetuses. This iso enzyme is supposed to be feto protective in humans. In the present study, we observed that a nonsynonymous substitution of *CYP3A7*, N192S, was driven to near fixation in all human samples and fixed in non-Africans. The N192S SNP occurs in a region close to the CYP3As substrate recognition site 2 (Xue et al. 2001). Alignments of amino acid sequences with all human CYP3A and the orthologs in rat (Cyp3a9) and mouse (Cyp3a13) by us indicated that the residue 192N is highly conserved (data not shown). It is possible that the N192S substitution affects the regioselectivity of CYP3A7 toward its substrates and thus is favored by natural selection. Interestingly, Rodríguez-Antona et al. (2005) recently reported that a common nonsynonymous variant CYP3A7*2 is in strong LD with CYP3A5*1. The CYP3A7*1/CYP3A5*3 haplotype is associated with CYP3A7.1 expression but without CYP3A5 expression, whereas the CYP3A7*2/CYP3A5*1 haplotype is associated with the expression of CYP3A5 and a more active form of CYP3A7 (Rodríguez-Antona et al. 2005). Unfortunately, however, we failed to identify the CYP3A7*2 variant in our study because of the highly homologous sequences of the *CYP3A* family, which prevented our addressing the possibility that CYP3A7*2 might be the target of positive selection.

One of the novel findings in our study was the unique natural selection pattern on *CYP3A43* in non-Africans. Most *CYP3A43* haplotypes in non-Africans contained the high frequency–derived alleles at G83448A and C83643T polymorphic sites. In addition, the CYP3A43*2 and CYP3A43*3 variants were also in significant LD with these SNPs in non-Africans. The skewed haplotype structure and significant result of haplotype diversity test suggest that the major haplotype in this region is favored by natural selection in non-Africans. The five variants that separated the two *CYP3A43* haplotype clusters had extremely high *F*_ST_ values for the Chinese–African and Caucasian–African comparisons, which also indicates the impact of population-specific selective pressures on *CYP3A43* in populations outside of Africa.

All neutrality tests showed significant departure from neutrality for the sequences from the *CYP3A5* exon 2 to the *CYP3A5* 3′ untranslated region in Caucasians. The remarkable interpopulation differences in both frequency spectrum and haplotype structure in *CYP3A5* also indicate a recent selective sweep in Caucasians, which is in accordance with the findings by Thompson et al. (2004). CYP3A5 is important in the metabolism of endogenous substrates such as cortisol and in environmental chemicals such as aflatoxin B_1_. CYP3A5*3 reportedly undergoes strong selective pressure (e.g., salt sensitivity) (Thompson et al. 2004). Other selective pressures, especially from dietary chemical exposure, may also play a role in shaping the unusual nucleotide diversity patterns in *CYP3A5* in Caucasians.

It has become clear that populations and individuals have their own individual finger-print of unique allelic architecture coding the xenobiotic response system, and these genetic variants have functional relevance with respect to drug response and environmentally related diseases (Nebert and Dieter 2000). Drug-metabolizing enzyme (DME) genes are among the most favorable targets of natural selection for their role in metabolism of environmental compounds. Recent genome-wide analyses indicate that the human xeno biotic metabolism and disposition system may have undergone unique natural selection (Sabeti et al. 2007). It has also been shown in candidate gene–based studies that positive selection has acted on DME genes such as *CYP1A2* (Wooding et al. 2002), *CYP3A4* and *CYP3A5* (Schirmer et al. 2006; Thompson et al. 2004), and *FMO3* (flavin-containing mono-oxygenase 3) (Allerston et al. 2007), all phase I DME genes; *NAT2* (*N*-acetyltransferase 2) (Patin et al. 2006) and *UGT2B17* (UDP-glucuronosyltransferase 2 family, polypeptide B17) (Xue et al. 2008), both phase II DME genes; *ABCB1* [ATP-binding cassette, subfamily B, member 1; also named multidrug resistance 1 (*MDR1*)] (Tang et al. 2004; Wang et al. 2007), and *ABCC1* (Wang et al. 2005), both phase III DMEs. Among these genes, *CYP3A* and *ABCB1* are very interesting outliers because of their unique genetic and biochemical properties. The *CYP3A* gene cluster is located at chromosome 7, just 119 kb away from the *ABCB1* locus, which codes MDR1 (P-glycoprotein), the best-characterized phase III drug transporter and one of the major determinants of the absorption, distribution, metabolism, and excretion/toxicologic profiles for a large range of hydro phobic exogenous substrates, including nearly every category of clinically important drugs, with a substrate spectrum similar to that of CYP3A enzymes. In the liver, lung, kidney, and intestine, there is a close correlation between the expressions of CYP3A4 and ABCB1 and their transcriptional factor, the pregnane X receptor (Miki et al. 2005), which coordinately regulates xenobiotic/drug metabolism and efflux through *trans*-activating the expression of the two genes (Synold et al. 2001). We and other groups have previously reported positive selection on functional derived variants of *ABCB1* in its coding and regulatory regions in human populations, which are correlated with increased expression and enhanced transporter activity (Tang et al. 2004; Wang et al. 2007). The selection hotspot on the two closely linked major xenobiotic response genes, *CYP3A* and *ABCB1*, superimposed with their closely linked genomic map, their finely coordinated *trans*-activation for xenobiotic disposition and clearance, and their overlapping tissue expression profile and substrate spectrum, may provide a good model in evolutionary environmental medicine to decipher the integral and dynamic profile of the organization, function, and evolution of the xenobiotic disposition system.

In summary, this evolutionary population genetics study on the human *CYP3A* locus confirmed the previously reported positive selection on *CYP3A5* in non-Africans, revealed that *CYP3A4* and *CYP3A7* are under a recent or ongoing positive natural selection in the general human population, and also pointed to positive natural selection on *CYP3A43* in non-Africans. The complex pattern of natural selection on the allelic structure of the *CYP3A* locus may underlie the phenotypic variation in CYP3A activity in populations from different geographic regions and having inter-ethnic differences in response to drugs and herbal medicine as well as ethnic differences in predisposition to complex disorders resulting from CYP3A-substrate exposure.

## Figures and Tables

**Table 1:** Nucleotide diversity and neutrality tests for the entire *CYP3A* locus.

Summary statistic	Chinese	Caucasians	Africans	Non-Africans	Overall
Sample size	29	24	24	48	77
No. of segregating sites	57	60	113	91	167
No. of singletons	32	29	42	42	74
π (× 10^−4^)	1.6	1.4	4.0	1.5	2.6
θ_W_ (× 10^−4^)	2.8	3.1	5.8	3.9	6.8
θ_H_ (× 10^−4^)	3.5	4.5	5.6	5.1	6.8
D%^a^	0.97	0.95	0.97	0.95	0.94
Tajima’s *D*	−1.47*	−1.96*	−1.084	−1.98*	−1.99*
Fu and Li’s D*	−3.20*	−2.21	−1.344	−4.55*	−11.10*
Fu and Li’s F*	−3.04*	−2.52*	−1.397	−3.97*	−8.48*
Fay and Wu’s *H*	−6.94	−13.81*	−5.42	−15.61	−16.24
HKA *p-*value	0.01**	0.01**	0.01**	0.01**	0.01**

a Fixed sequence differences between human and chimpanzee.

* *p* < 0.05.

** *p* < 0.01.

**Table 2:** Haplotype diversity test in *CYP3A43* and *CYP3A5* gene regions.

	*CYP3A43*		*CYP3A5*	
Population	Haplotype diversity	*p*-Value	Haplotype diversity	*p*-Value
Chinese	0.257	0.002	0.547	0.226
Caucasian	0.373	0.008	0.467	0.038
African	0.860	0.531	0.801	0.663
Non-African	0.310	0.004	0.518	0.055
Overall	0.533	0.006	0.662	0.076

## References

[b1-ehp-117-1541] Abecasis GR, Cookson WO (2000). GOLD—graphical overview of linkage disequilibrium. Bioinformatics.

[b2-ehp-117-1541] Allerston CK, Shimizu M, Fujieda M, Shephard EA, Yamazaki H, Phillips IR (2007). Molecular evolution and balancing selection in the flavin-containing monooxygenase 3 gene (FMO3). Pharmacogenet Genomics.

[b3-ehp-117-1541] Bamshad M, Wooding SP (2003). Signatures of natural selection in the human genome. Nat Rev Genet.

[b4-ehp-117-1541] Bandelt HJ, Forster P, Rohl A (1999). Median-joining networks for inferring intraspecific phylogenies. Mol Biol Evol.

[b5-ehp-117-1541] Bowcock AM, Kidd JR, Mountain JL, Hebert JM, Carotenuto L, Kidd KK (1991). Drift, admixture, and selection in human evolution: a study with DNA polymorphisms. Proc Natl Acad Sci USA.

[b6-ehp-117-1541] Burk O, Tegude H, Koch I, Hustert E, Wolbold R, Glaeser H (2002). Molecular mechanisms of polymorphic CYP3A7 expression in adult human liver and intestine. J Biol Chem.

[b7-ehp-117-1541] Center for Statistical Genetics (2009). GOLD: Graphical Overview of Linkage Disequilibrium.

[b8-ehp-117-1541] Chu JY, Huang W, Kuang SQ, Wang JM, Xu JJ, Chu ZT (1998). Genetic relationship of populations in China. Proc Natl Acad Sci USA.

[b9-ehp-117-1541] Dabney A, Storey J (2008). Q-VALUE.

[b10-ehp-117-1541] Di Rienzo A, Hudson RR (2005). An evolutionary framework for common diseases: the ancestral-susceptibility model. Trends Genet.

[b11-ehp-117-1541] Ding K, Zhou K, He F, Shen Y (2003). LDA—a java-based linkage disequilibrium analyzer. Bioinformatics.

[b12-ehp-117-1541] Dorne JL, Walton K, Renwick AG (2003). Human variability in CYP3A4 metabolism and CYP3A4-related uncertainty factors for risk assessment. Food Chem Toxicol.

[b13-ehp-117-1541] Ebersberger I, Metzler D, Schwarz C, Paabo S (2002). Genome-wide comparison of DNA sequences between humans and chimpanzees. Am J Hum Genet.

[b14-ehp-117-1541] Fay JC, Wu CI (2000). Hitchhiking under positive Darwinian selection. Genetics.

[b15-ehp-117-1541] Finta C, Zaphiropoulos PG (2000). The human cytochrome P450 3A locus. Gene evolution by capture of downstream exons. Gene.

[b16-ehp-117-1541] Fluxus Technology (2008). Free Phylogenetic Network Software.

[b17-ehp-117-1541] Franklin MR, Phillips JD, Kushner JP (2000). CYP3A-inducing agents and the attenuation of uroporphyrin accumulation and excretion in a rat model of porphyria cutanea tarda. Biochem Pharmacol.

[b18-ehp-117-1541] Fu YX, Li WH (1993). Statistical tests of neutrality of mutations. Genetics.

[b19-ehp-117-1541] Fujita K (2004). Food-drug interactions via human cytochrome P450 3A (CYP3A). Drug Metabol Drug Interact.

[b20-ehp-117-1541] Fullerton SM, Bartoszewicz A, Ybazeta G, Horikawa Y, Bell GI, Kidd KK (2002). Geographic and haplotype structure of candidate type 2 diabetes susceptibility variants at the calpain-10 locus. Am J Hum Genet.

[b21-ehp-117-1541] Gellner K, Eiselt R, Hustert E, Arnold H, Koch I, Haberl M (2001). Genomic organization of the human CYP3A locus: identification of a new, inducible CYP3A gene. Pharmacogenetics.

[b22-ehp-117-1541] Givens RC, Lin YS, Dowling AL, Thummel KE, Lamba JK, Schuetz EG (2003). CYP3A5 genotype predicts renal CYP3A activity and blood pressure in healthy adults. J Appl Physiol.

[b23-ehp-117-1541] Jin L, Su B (2000). Natives or immigrants: modern human origin in east Asia. Nat Rev Genet.

[b24-ehp-117-1541] Kaplan N, Hudson RR, Iizuka M (1991). The coalescent process in models with selection, recombination and geographic subdivision. Genet Res.

[b25-ehp-117-1541] Keshava C, McCanlies EC, Weston A (2004). CYP3A4 polymorphisms—potential risk factors for breast and prostate cancer: a HuGE review. Am J Epidemiol.

[b26-ehp-117-1541] Kuehl P, Zhang J, Lin Y, Lamba J, Assem M, Schuetz J (2001). Sequence diversity in CYP3A promoters and characterization of the genetic basis of polymorphic CYP3A5 expression. Nat Genet.

[b27-ehp-117-1541] Lee AJ, Conney AH, Zhu BT (2003). Human cytochrome P450 3A7 has a distinct high catalytic activity for the 16α-hydroxylation of estrone but not 17β-estradiol. Cancer Res.

[b28-ehp-117-1541] Leeder JS, Gaedigk R, Marcucci KA, Gaedigk A, Vyhlidal CA, Schindel BP (2005). Variability of CYP3A7 expression in human fetal liver. J Pharmacol Exp Ther.

[b29-ehp-117-1541] Marill J, Capron CC, Idres N, Chabot GG (2002). Human cytochrome P450s involved in the metabolism of 9- *cis*- and 13-*cis*-retinoic acids. Biochem Pharmacol.

[b30-ehp-117-1541] Miki Y, Suzuki T, Tazawa C, Blumberg B, Sasano H (2005). Steroid and xenobiotic receptor (SXR), cytochrome P450 3A4 and multidrug resistance gene 1 in human adult and fetal tissues. Mol Cell Endocrinol.

[b31-ehp-117-1541] Nachman MW, Crowell SL (2000). Contrasting evolutionary histories of two introns of the Duchenne muscular dystrophy gene, *Dmd*, in humans. Genetics.

[b32-ehp-117-1541] National Center for Biotechnology Information (2009). GenBank Overview.

[b33-ehp-117-1541] Nebert DW, Dieter MZ (2000). The evolution of drug metabolism. Pharmacology.

[b34-ehp-117-1541] Ohmori S, Nakasa H, Asanome K, Kurose Y, Ishii I, Hosokawa M (1998). Differential catalytic properties in metabolism of endogenous and exogenous substrates among CYP3A enzymes expressed in COS-7 cells. Biochim Biophys Acta.

[b35-ehp-117-1541] Patin E, Barreiro LB, Sabeti PC, Austerlitz F, Luca F, Sajantila A (2006). Deciphering the ancient and complex evolutionary history of human arylamine *N*-acetyltransferase genes. Am J Hum Genet.

[b36-ehp-117-1541] Qiu H, Taudien S, Herlyn H, Schmitz J, Zhou Y, Chen G (2008). CYP3 phylogenomics: evidence for positive selection of CYP3A4 and CYP3A7. Pharmacogenet Genomics.

[b37-ehp-117-1541] Rodríguez-Antona C, Jande M, Rane A, Ingelman-Sundberg M (2005). Identification and phenotype characterization of two CYP3A haplotypes causing different enzymatic capacity in fetal livers. Clin Pharmacol Ther.

[b38-ehp-117-1541] Rozas J, Sanchez-DelBarrio JC, Messeguer X, Rozas R (2003). DnaSP, DNA polymorphism analyses by the coalescent and other methods. Bioinformatics.

[b39-ehp-117-1541] Sabeti PC, Varilly P, Fry B, Lohmueller J, Hostetter E, Cotsapas C (2007). Genome-wide detection and characterization of positive selection in human populations. Nature.

[b40-ehp-117-1541] Sachidanandam R, Weissman D, Schmidt SC, Kakol JM, Stein LD, Marth G (2001). A map of human genome sequence variation containing 1.42 million single nucleo tide polymorphisms. Nature.

[b41-ehp-117-1541] Schirmer M, Toliat MR, Haberl M, Suk A, Kamdem LK, Klein K (2006). Genetic signature consistent with selection against the *CYP3A4*1B* allele in non-African populations. Pharmacogenet Genomics.

[b42-ehp-117-1541] Schuetz JD, Kauma S, Guzelian PS (1993). Identification of the fetal liver cytochrome CYP3A7 in human endometrium and placenta. J Clin Invest.

[b43-ehp-117-1541] Siiteri PK, MacDonald PC (1963). The utilization of circulating dehydroepiandrosterone sulfate for estrogen synthesis during human pregnancy. Steroids.

[b44-ehp-117-1541] Snider NT, Kornilov AM, Kent UM, Hollenberg PF (2007). Anandamide metabolism by human liver and kidney microsomal cytochrome p450 enzymes to form hydroxyeicosatetraenoic and epoxyeicosatrienoic acid ethanolamides. J Pharmacol Exp Ther.

[b45-ehp-117-1541] Stephens JC, Schneider JA, Tanguay DA, Choi J, Acharya T, Stanley SE (2001). Haplotype variation and linkage disequilibrium in 313 human genes. Science.

[b46-ehp-117-1541] Stephens M, Donnelly P (2003). A comparison of Bayesian methods for haplotype reconstruction from population genotype data. Am J Hum Genet.

[b47-ehp-117-1541] Storey JD, Tibshirani R (2003). Statistical significance for genome-wide studies. Proc Natl Acad Sci USA.

[b48-ehp-117-1541] Storz JF, Payseur BA, Nachman MW (2004). Genome scans of DNA variability in humans reveal evidence for selective sweeps outside of Africa. Mol Biol Evol.

[b49-ehp-117-1541] Synold TW, Dussault I, Forman BM (2001). The orphan nuclear receptor SXR coordinately regulates drug metabolism and efflux. Nat Med.

[b50-ehp-117-1541] Tajima F (1989). Statistical method for testing the neutral mutation hypothesis by DNA polymorphism. Genetics.

[b51-ehp-117-1541] Tang K, Wong LP, Lee EJ, Chong SS, Lee CG (2004). Genomic evidence for recent positive selection at the human MDR1 gene locus. Hum Mol Genet.

[b52-ehp-117-1541] Thompson EE, Kuttab-Boulos H, Witonsky D, Yang L, Roe BA, Di Rienzo A (2004). CYP3A variation and the evolution of salt-sensitivity variants. Am J Hum Genet.

[b53-ehp-117-1541] van Waterschoot RA, Rooswinkel RW, Wagenaar E, van der Kruijssen CM, van Herwaarden AE, Schinkel AH (2009). Intestinal cytochrome P450 3A plays an important role in the regulation of detoxifying systems in the liver. FASEB J.

[b54-ehp-117-1541] Wang H, Ding K, Zhang Y, Jin L, Kullo IJ, He F (2007). Comparative and evolutionary pharmacogenetics of ABCB1: complex signatures of positive selection on coding and regulatory regions. Pharmacogenet Genomics.

[b55-ehp-117-1541] Wang Z, Wang B, Tang K, Lee EJ, Chong SS, Lee CG (2005). A functional polymorphism within the MRP1 gene locus identified through its genomic signature of positive selection. Hum Mol Genet.

[b56-ehp-117-1541] Watterson GA (1975). On the number of segregating sites in genetical models without recombination. Theor Popul Biol.

[b57-ehp-117-1541] Weir BS, Cockerham CC (1984). Estimating F-statistics for the population structure. Evolution.

[b58-ehp-117-1541] Wojnowski L (2004). Genetics of the variable expression of CYP3A in humans. Ther Drug Monit.

[b59-ehp-117-1541] Wooding SP, Watkins WS, Bamshad MJ, Dunn DM, Weiss RB, Jorde LB (2002). DNA sequence variation in a 3.7-kb non-coding sequence 5’ of the CYP1A2 gene: implications for human population history and natural selection. Am J Hum Genet.

[b60-ehp-117-1541] Xue L, Wang HF, Wang Q, Szklarz GD, Domanski TL, Halpert JR (2001). Influence of P450 3A4 SRS-2 residues on cooperativity and/or regioselectivity of aflatoxin B_1_ oxidation. Chem Res Toxicol.

[b61-ehp-117-1541] Xue Y, Sun D, Daly A, Yang F, Zhou X, Zhao M (2008). Adaptive evolution of UGT2B17 copy-number variation. Am J Hum Genet.

[b62-ehp-117-1541] Zawaira A, Matimba A, Masimirembwa C (2008). Prediction of sites under adaptive evolution in cytochrome P450 sequences and their relationship to substrate recognition sites. Pharmacogenet Genomics.

[b63-ehp-117-1541] Zhang K (2004). HaploBlockFinder V0.7.

[b64-ehp-117-1541] Zhang K, Jin L (2003). HaploBlockFinder: haplotype block analyses. Bioinformatics.

[b65-ehp-117-1541] Zhou SF, Xue CC, Yu XQ, Wang G (2007). Metabolic activation of herbal and dietary constituents and its clinical and toxicological implications: an update. Curr Drug Metab.

